# The Intervention Areas of the Psychologist in Pediatric Palliative Care: A Retrospective Analysis

**DOI:** 10.3389/fpsyg.2022.858812

**Published:** 2022-03-22

**Authors:** Anna Santini, Irene Avagnina, Anna Marinetto, Valentina De Tommasi, Pierina Lazzarin, Giorgio Perilongo, Franca Benini

**Affiliations:** ^1^Pediatric Pain and Palliative Care Service, Department of Women’s and Children’s Health, School of Medicine and Surgery, University of Padua, Padua, Italy; ^2^Department of Women’s and Children’s Health, School of Medicine, University of Padua, Padua, Italy

**Keywords:** pediatric palliative care (PPC), retrospective analysis, biopsychosocial model, intervention areas, psychologist

## Abstract

Infants, children and adolescents with life-limiting and life-threatening disease need long-term care that may change according to disease’s natural history. With the primary goal of quality of life, the psychologist of pediatric palliative care (PPC) network deals with a large variety of issues. Little consideration has been given to the variety of intervention areas of psychology in PPC that concern the whole life span of the patient and family. The PPC network is composed by a multidisciplinary team of palliative care specialists that intervenes at home, in the hospital and in every place where the patient is living. The network coordinates different public health services to respond to clinical, psychosocial and spiritual needs. In these scenarios, the psychological need is not a single event but a moment inserted in the complexity of the child’s needs. This retrospective monocentric project consists of an analysis of characteristics of psychological interventions in our PPC service. The time frame taken into consideration is 2019–2020, analyzing the clinical records of 186 patients of Pediatric Palliative Care and Pain Service of Veneto Region (Italy). The areas that emerged in the analysis show how the intervention of the psychologist in PPC does not concern only end-of-life, but a series of topics that are significant for the family to guarantee psycho-social wellbeing oriented toward the best quality of life. In conclusion, these different topics highlight the complexity of the child and family experience. This variety must be taken into consideration, the psychologist must increase holistic support with a dedicated skills curriculum.

## Introduction

Palliative care for children and adults is defined by the World Health Organization as the active and global care of the child with life limiting and life threatening diseases and his family. This care involves managing the child and families’ physical, psychosocial and spiritual needs (World Health Organization [WHO], 2018). The criteria for eligibility for pediatric palliative care (PPC) primarily include the presence of a diagnosis of life limiting and life threatening diseases and a highly complex care situation (Benini et al., 2008). PPC are provided by a multidisciplinary specialist team (Fitchett et al., 2011; O’Connor and Fisher, 2011; Verberne et al., 2017; Cervantes Blanco and Jones, 2018) and interface with the professionals present in the other health centers. The network is a functional and integrated aggregation of PPC activities delivered in hospital, pediatric hospice and at home. It is made up of all the institutions necessary for the child care management (hospital and territorial health, socio-health, social-assistance and educational services) and integrates and collaborates with the networks, pediatric and not, existing in the geographic area (Bergstraesser et al., 2013). The psychologist is part of this specialist team which aims to respond in a synergistic way to global needs that are taken care of in a biopsychosocial paradigm (Quill et al., 2003; Brown, 2006; Moore, 2019; Ribbers et al., 2020; Benini et al., 2022).

The experience of the caregivers of young patients is widely taken into consideration. The researchers underline patients’ family members’ fatigue and different psychosocial needs (Jünger et al., 2010a; Collins et al., 2016; Perpiñá-Galvañ et al., 2019) who often live with a strong sense of guilt (Gonyea et al., 2008). The impact of being a caregiver involves social isolation and work difficulties (Wakabayashi and Donato, 2005; Knapp et al., 2010; Price et al., 2012; Collins et al., 2016; Harputluoğlu et al., 2021). Stress, anxiety and depression are common in parents (Collins et al., 2016; Wightman, 2020) and can generate traumatic experiences (Mitchell et al., 2006; Kars et al., 2011). Such emotions are present not only in adult caregivers but also in pediatric patients and their siblings (Barlow and Ellard, 2006; Collins et al., 2016; Weaver et al., 2019).

Family’s reaction mechanisms are a promoter of the child’s health (Cipolletta et al., 2015) and this allows them to develop coping and emotional management strategies (Sloper, 2000; Goldbeck, 2006; Felipe Silva et al., 2021). Psychological needs can be traced throughout the disease’s history, which can be very long and can have different critical moments (Mitchell et al., 2006; Liben et al., 2008). Caregivers often feel they are in a battle and use the metaphor of “combat” to describe their story (Knapp et al., 2010), so continuous monitoring of psychosocial needs is crucial (Goldbeck, 2006; Mitchell et al., 2006; Liben et al., 2008; Weaver et al., 2016). The PPC psychologist takes care not only of the patient, but of the entire family (Kazak and Noll, 2015).

Pediatric palliative care is not limited to end-of-life support, but involves the whole natural history of child’s disease, even where specific treatments for the disease are pursued (Nilsson et al., 2020).

Despite this, PPC is often associated only with end-of-life care and this leads many professionals to not use the service adequately or refer patients late (Thompson et al., 2009; Twamley et al., 2014). Professionals show a certain reluctance to send families to CPP (Davies et al., 2008; Knapp and Thompson, 2012) both because they struggle to use certain terms with their parents (Davies et al., 2008; Durall et al., 2012) and because families can perceive this as a giving up by lead physicians (Thompson et al., 2009).

Furthermore, there is a lack of knowledge on the subject of PPC and this leads to little knowledge of temporal indicators, protocol and strategies (Thompson et al., 2009). In evaluating the specific challenges that differentiate CPP from adult CP, the following are pointed out: the need to offer family support to the whole family and not just to the patient; the presence of pathologies that can have very long life expectancies; the difficulty of having a health system that meets the special needs and priorities of children; the variety of care needs of different clinical conditions; the difficulty in relating not with the patient but often with family members and the tensions that arise; the priority of managing communications taking into account the parental authority and the communicative possibilities of the young patient; the need for highly specialized skills and knowledge (Jünger et al., 2010b). However, some works are also focusing more professional attention on the PPC issue and more recognition of PPC services in ensuring the patient’s quality of life (Jünger et al., 2010b; Bergstraesser et al., 2013).

The purpose of this work is to show, through the retrospective study of clinical diaries, the complexity of the psychologist’s work within the CPP network, in order to increase knowledge of a holistic and global care model.

## Methods

The sample examined was 225 patients, of which 57 were excluded due to private psychological counseling or absence of psychological sessions records at the time of the study. Therefore the sample of the clinical reports analyzed was 168 patients, of which 49 died. The time range taken into consideration runs from 01/01/2019 to 12/31/2020. The service had a number of variable psychologists (3 between 01/01/2019 to 01/07/2019, 2 between 02/07/2019 to 01/10/2020, and 1 between 02/10/2020 and 31/12/2020). In addition, this period also includes the beginning of the COVID-19 emergency, for which the activity of our service has seen a change since 07.03.2020.

The analysis objectives were to identify: the different teams with which the psychologist works during communications, the settings and target of psychological interviews, the interviews’ topics.

The material examined includes clinical reports, consultations and clinical charts for a total of 1292 interventions: 511 interventions by the PPC team and 781 psychological sessions. The psychological work has been divided on the basis of a thematic analysis (Vaismoradi et al., 2013; Nowell et al., 2017) which has made it possible to identify homogeneous thematic areas by topic. This process took place following the indications of Nowell et al. (2017), using the clinical material produced by psychologists. A deductive approach was used, the writings were analyzed using *a priori* categories relating to the psychological issues found in the literature and compared with the clinical experience of the PPC psychologists.

These categories have been discussed numerous times on the basis of the readings of clinical material, two different operators have worked on databases (authors AM, AS) and all discrepancies and uncertainties have been addressed through a peer-debriefing.

The thematic areas identified are:

• “Medical stress and symptoms management”: needs to build, modify or emotionally manage issues related to the disease and its symptoms.
• “Functional loss or device introduction”: critical issues and strategies of intervention focused on adaptation to a disability increase resulting from a function’s loss (e.g., mobility, sight, urination) or to a new medical device introduction (e.g., ventilator, wheelchair, tracheo).
• “Parenting and relationship”: issues related to practical and emotional management of significant family relationships but also formal (e.g., school, work) and informal network (e.g., friends).
• “Emotional displays”: manage expressions of emotions (e.g., anxiety, fear, depression, joy).
• “Behavioral manifestations”: behavioral expressions that are considered critical for some reason (e.g., hyperactivity, impulse management difficulties).
• “Existential concerns”: thoughts related to the signification of events, the purpose of existence and anticipations of the future.
• “End of Life”: issues related to the critical moment that has been communicated at that time or that is glimpsed in the near future.
• “Advance care planning”: thoughts and experiences related to a decision on the proportionality of care for the patient’s disease.
• “Management covid pandemic period”: thoughts and experiences related to the management covid emergency.

## Results

Socio-demographic variables (Table 1) showed a patient average age of 9.5 years, mostly male (54.1%). The most frequent disease was non-oncological one (79.76%). Parents spoke fluent Italian for 88.10% of the sample, most of them were also a married couple (90.48%). In these families, another psychologist was rarely already present before accessing PPC service (6.19%).

**TABLE 1:** Socio-demographic variables.

Socio-demographic variables (tot = 168)	
Mean age, years (st. deviation)	9,49 (± 6)
Male, number (%)	91 (54,17%)
**Type of disease, number (%)**	
Oncological	27 (16,07%)
Non-oncological	134 (79,76%)
Undefined diagnosis*	7 (4,17%)
Fluent italian, number (%)	148 (88,10%)
**Parents couple status, number (%)**	
Unite	152 (90,48%)
divorced	13 (7,74%)
Widow/-er	1 (0,60%)
Foster family	2 (1,19%)
Presence of other psychologist, number (%)	44 (6,19%)

**FIGURE 1:**
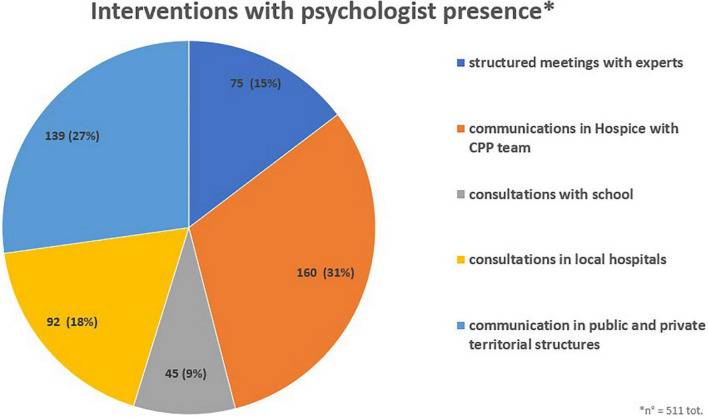
Interventions with psychologist.

As interventions with psychologist presence were all communications that involve psychologists with other members of the PPC team (Figure 1). The highest number of communications were in Hospice with members of the CPP team (31.3%) such as communications relating to treatment, worsening clinical conditions, end-of-life communications, etc. Other interventions included discussions with public and private territorial structures (27.2%) with the aim of sharing family needs and coordinating resources. The PPC team also carried out consultations in local hospitals (18.0%). A structured and formalized intervention was carried by a multidimensional assessment unit to define an individualized care project. Furthermore, the PPC psychologist carried out information and training meetings for school staff (8.8%).

A total of 781 psychological sessions were carried out and divided on the basis of target (Table 2). Most of the interventions were carried out with caregivers (63.5%) and then with patients (18.4%). Less frequently, psychological sessions were carried out with the couple (8.3%), siblings (4.7%), entire family unit (2.7%), and extended family (2.3%). In general, the most addressed issues were relationship management (26.50%) and emotional manifestations (25.22%). Specifically for the target, the parent’s sessions concerned manifestations management (43.75%) and relationships management (20.83%). Caregivers, on the other hand, mostly dealt with parenting and relationships (31.45%) and emotional variables (19.56%) management. Couple sessions mainly concerned end-of-life (47.69%). The end-of-life topic latter issue was more commonly addressed in interviews with the extended family (38.89%), while the family in the strict sense mainly managed interpersonal relationships (33.33%). Siblings, instead, dealt more with emotional management (51.35%).

**TABLE 2:** Thematic areas.

Thematic areas							

	**Patient, *n* (%)**	**Caregiver, *n* (%)**	**Parents, *n* (%)**	**Siblings, *n* (%)**	**Family, *n* (%)**	**Extended family, *n* (%)**	**Total, *n* (%)**
Medical stress and symptoms management	27 (18,75%)	72 (14,52%)	7 (10,77%)	1 (2,70%)	2 (9,52%)	1 (5,56%)	110 (14,08%)
Functional loss or device introduction	7 (4,86%)	29 (5,85%)	1 (1,54%)	0 (0%)	0 (0%)	0 (0,00%)	37 (4,74%)
Parenting and relationship	30 (20,83%)	156 (31,45%)	8 (12,31%)	4 (10,81%)	7 (33,33%)	2 (11,11%)	207 (26,50%)
Emotional displays	63 (43,75%)	97 (19,56%)	9 (13,85%)	19 (51,35%)	3 (14,29%)	6 (33,33%)	197 (25,22%)
Behavioral manifestations	4 (2,78%)	3 (0,60%)	1 (1,54%)	2 (5,41%)	4 (19,05%)	0 (0,00%)	14 (1,79%)
Existential concerns	8 (5,56%)	42 (8,47%)	5 (7,69%)	2 (5,41%)	0 (0,00%)	2 (11,11%)	59 (7,55%)
EoL	0 (0%)	55 (11,09%)	31 (47,69%)	9 (24,32%)	4 (19,05%)	7 (38,89%)	106 (13,57%)
Advance care planning	0 (0%)	7 (1,41%)	3 (4,62%)	0 (0%)	0 (0,00%)	0 (0,00%)	10 (1,28%)
Management covid	5 (3,47%)	35 (7,06%)	0 (0%)	0 (0%)	1 (4,76%)	0 (0,00%)	41 (5,25%)
Total	144 (18,44%)	496 (63,51%)	65 (8,32%)	37 (4,74%)	21 (2,69%)	18 (2,30%)	781

In general (Table 3), the most common setting was hospice (52.5%), followed by sessions conducted online (27.7%) and by those at home (14.6%). This particular covid-emergency period may had led to favoring online sessions. The least used setting was the hospital (5.12%). Although the hospice setting was the most common, the out-of-hospital setting (home and online) was used for 43.75% by patients, for caregivers for 46.37%, for couples 20%, for siblings 37.84%, for the family 47.62% and for the extended family it was only 5.56%.

**TABLE 3:** Psychological sessions setting.

Setting							

	**Patient, *n* (%)**	**Caregiver, *n* (%)**	**Parents, *n* (%)**	**Siblings, *n* (%)**	**Family, *n* (%)**	**Extended family, *n* (%)**	**Total, *n* (%)**
Home	34 (23,61%)	45 (9,07%)	10 (15,38%)	14 (37,84%)	10 (47,62%)	1 (5,56%)	114 (14,60%)
Hospital	5 (3,47%)	25 (5,04%)	6 (9,23%)	1 (2,70%)	1 (4,76%)	2 (11,11%)	40 (5,12%)
Hospice	76 (52,78%)	241 (48,59%)	46 (70,77%)	22 (59,46%)	10 (47,62%)	15 (83,33%)	410 (52,50%)
Online	29 (20,14%)	185 (37,30%)	3 (4,62%)	0 (0,00%)	0 (0,00%)	0 (0,00%)	217 (27,78%)
Total	144 (18,44%)	496 (63,51%)	65 (8,32%)	37 (4,74%)	21 (2,69%)	18 (2,30%)	781

## Discussion

### Teams

The psychologist is one part of a PPC professional’s network, because of this a large number of communications is given in the presence of some team members based on the purposes (31.31%, n = 160). In addition, the psychologist as part of PPC is present in the communications that are given both with hospital and territorial professionals networks (68.69, n = 351). The psychologist is inserted during communications with the aim of being a facilitator of communication and witness to continue interviews later with the family and the child. The PPC psychologist participates in communications with the aim of being communication facilitator and witness to continue the psychological work later.

### Settings

Our results show a flexibility of the psychologist’s work with regard to the setting that could be home or online (42.38%) even if a higher percentage of interviews is conducted in Hospice (52.5%). Hospice, as a clinical setting for follow-up, is where evaluation and diagnostic assessment take place.

However, when the family returns home the psychologist enters the family routine. The many sessions conducted online are probably increased due to the historical moment of the covid pandemic, even so these are effective means of structuring the therapeutic alliance (Lai et al., 2020).

### Target and Content Analysis

These results show that psychological interviews targets are mainly caregivers (63.51%) and it is less frequent that they are patients (18.44%). This data can be explained by the fact that children in PPC may have various cognitive disabilities that do not allow them to have a proper psychological session. Furthermore, the average age of the sample is 9 and a half years, with a standard deviation of 6, which is why not all patients are currently able to have a psychological session. However, parents are a common target of psychological sessions (Strada and Sourkes, 2009), but it is crucial to analyze a child’s cognitive and understanding abilities. The child always has the right to know his situation and express his wishes (Benini et al., 2014b).

Overall, the prevailing themes are management relationships (26.5%), emotions (25.22%) and medical stress (14.08%). These results are in line with some research in the literature that shows that negative emotions such as anxiety, fear, depression are very present in patients and families (Mitchell et al., 2006; Collins et al., 2016).

The management of uncertainty becomes the norm and allows us to create coping mechanisms to deal with new problems and critical issues (Santini et al., 2020). Part of the psychologist’s work is to stimulate family resilience. The changes also affect the quality and quantity of relationships in different environments (e.g., work for parents and school for children). Children in CPP have a very variable life expectancy and unpredictable disease course, which is why they face many psychological and social challenges.

If patients need to talk mainly about their emotions (43.75%), parents are more focused on how to relate to their children and to the formal and informal network. From a systemic perspective, the meetings with both parents focused on end-of-life management and the sessions with extended family also had this topic. The importance of being able to discuss the future and the difficult moment of the death of a child emerges, the role of the psychologist here is to allow the sharing of thoughts, emotions, memories and wishes. Managing everyday family life can become difficult after bad news, it can be equally difficult to enjoy the present moments without thinking about death (Lou et al., 2015; Verberne et al., 2019).

It is interesting that siblings have dealt above all with the emotional part, perhaps because brothers are often put in a position to witness complex situations that are not always explained to them and at the same time they develop greater maturity and independence (Gaab et al., 2014). However, greater attention and space should be given to siblings’ personal experiences.

## Limitation

This work shows the variety of the psychologist’s skills in CPP, but the main research limitations concerning database. The clinical records are certainly not as punctual as verbatim transcripts and not all contacts between the family and the psychologist have been officially documented. Interviews cover the history of the disease, but it is interesting for future work to analyze the psychologist role after patient death. The data collected can be a first exploration, it would be interesting to be able to evaluate also the effectiveness of the interventions or analyze drop-outs. It might be interesting to consider the perspective of the patient and the family through a qualitative analysis of their point of view. Other future interventions could be aimed at understanding which interventions were useful and why.

## Conclusion

The management of complexity is the mission of PPC professionals, the psychologist is called to improve transversal skills and specific intervention methods. The psychologist must first of all show flexibility, combined with the ability to 1dialog with all professionals and be able to deal with different topics with changing targets. Health is promoted in different places of care, which are not only the hospital environment.

In this regard, a dedicated skills curriculum has been developed in the Italian PPC (Benini et al., 2014a). The main characteristics that are required of the psychologist working in this field are:

- ability to analyze psychosocial characteristics and potential issues of clinical situation;
- ability to structure an emotionally meaningful relationship with the child and his/her family members aimed at orienting the team’s work to the most appropriate communication methods;
- ability to carry out a clinical evaluation and psychological intervention projects aimed at children, couples and families in the developmental phase of the disease, in situations of chronic pain and in the proximity of death and bereavement;
- ability to integrate one’s specific professional contribution within the team work in order to promote awareness of the emotional and relational dimension in the working group;
- awareness of one’s own emotional sphere and personal mechanisms for constant self-observation;
- organizational and administrative skills necessary to contribute to the development, institutional/territorial rooting and management of specialisti PPC networks;
- continuous training on methods and intervention techniques and ability to manage research projects.

In general, it is necessary to think of a clear training path for the psychologist in PPC because this expert is called upon to operate in extremely complex areas.

## Data Availability Statement

The original contributions presented in the study are included in the article/supplementary material, further inquiries can be directed to the corresponding author.

## Author Contributions

AM, AS, and VD were involved in the data recollection and thematic analysis. IA and AS drafted the manuscript. PL, GP, and FB were involved in the critical revision of the manuscript and approved the final version of the manuscript. All authors contributed to the article and approved the submitted version.

## Conflict of Interest

The authors declare that the research was conducted in the absence of any commercial or financial relationships that could be construed as a potential conflict of interest.

## Publisher’s Note

All claims expressed in this article are solely those of the authors and do not necessarily represent those of their affiliated organizations, or those of the publisher, the editors and the reviewers. Any product that may be evaluated in this article, or claim that may be made by its manufacturer, is not guaranteed or endorsed by the publisher.
